# Flour for Home Baking: A Cross-Sectional Analysis of Supermarket Products Emphasising the Whole Grain Opportunity

**DOI:** 10.3390/nu12072058

**Published:** 2020-07-10

**Authors:** Jaimee Hughes, Verena Vaiciurgis, Sara Grafenauer

**Affiliations:** 1 Grains & Legumes Nutrition Council, Mount Street, North Sydney, NSW 2060, Australia; j.hughes@glnc.org.au; 2 School of Medicine, University of Wollongong, Northfields Avenue, Wollongong, NSW 2522, Australia; vv849@uowmail.edu.au

**Keywords:** whole grain, dietary fibre, sodium, sustainability, gluten free

## Abstract

Flour, typically derived from wheat, rye, corn and rice is a pantry staple, providing structure to bread and baked goods. This study aimed to provide a cross-sectional analysis of flour for home baking, highlighting the nutrition composition of whole grain flour and identifying novel categories. An audit was undertaken in February 2020, in four major supermarkets in metropolitan Sydney (Aldi, Coles, IGA and Woolworths). Ingredient lists, Nutrition Information Panel, claims, and country of origin were collected. The median and range were calculated for energy, protein, fat, saturated fat, carbohydrate, sugars, dietary fibre and sodium. Overall, 130 products were collected, including 26 plain flour, 12 self-raising, 17 plain wholemeal, 4 wholemeal self-raising, 20 bread-making mixes (4 were whole grain), 20 other refined grain (including corn and rice flour), 17 gluten-free, 3 legume, 4 fruit/vegetable, 4 coconut and 3 other non-grain (e.g., hemp seed, cricket flour) products. Plain wheat flour dominated the category, while whole grain (wholemeal) made up 19% of products, yet they contained significantly more dietary fibre (*p* < 0.001) and protein (*p* < 0.001). Self-raising flours were significantly higher in sodium (*p* < 0.001) and gluten-free products were lower in protein and dietary fibre, making legume, buckwheat and quinoa flour a better choice. Sustainability principles in fruit and vegetable production and novel insect products have driven new product development. There is a clear opportunity for further on-pack promotion of whole grain and dietary fibre within the category via food product labelling.

## 1. Introduction

According to Food Standards Australia New Zealand (FSANZ), flours are defined as ‘products of grinding or milling of cereals, legumes or other seeds’ [1]. Flour lends structure to baked goods, like cakes, biscuits, pastry and bread, with a range in protein content to suit the purpose and desired outcome. Higher-protein flours provide a greater proportion of gluten and a stronger dough for products like bread and the reverse is true for use in cakes and biscuits. The flour category is primarily focused on wheat flour as plain flour (also referred to as all-purpose flour) or self-raising flour with added raising agents, either milled as white flour (utilising just the endosperm of the grain) or whole wheat or wholemeal flour (which includes all parts of the grain) for use in home baking. Although definitions vary internationally, in Australia, wholemeal is considered whole grain within the FSANZ definition—‘the intact grain or the dehulled, ground, milled, cracked or flaked grain where the constituents—endosperm, germ and bran—are present in such proportions that represent the typical ratio of those fractions occurring in the whole cereal, and includes wholemeal’ [2]. 

Flour is a universal pantry staple, supplied as part of rations in the early colony of Australia, with as little as 2 1/2 pounds (1.3 kg) supplied in the ‘hungry years’ (1788–1792), and up to ten pounds (4.5 kg) in the 1830s [3]. Whereas bread remains a core food within the Australian diet, and bread-making flour is fortified with nutrients such as thiamin, folate and iodine [4], often, the final product from baking would be considered a discretionary food. Discretionary foods like cakes, biscuits and pastries are usually high in saturated fat, sugar or salt, and low in dietary fibre and micronutrients, and should be consumed infrequently and in small amounts in alignment with the Australian Dietary Guidelines [5]. From the National Nutrition and Physical Activity Survey 2011–2012 (NNPAS) [6], discretionary foods have been highlighted as a concern in Australia, with an average of 35% of energy derived from these non-core food and beverage choices. When considering the contribution of energy (E) from discretionary flour-based items like cakes, muffins, scones and cake-type desserts (3.4% of E), pastries (2.6% of E), sweet and savoury biscuits (2.5% of E), together, these foods contribute almost 25% of the energy derived from discretionary food, and these foods are consumed across all age groups. 

Data suggest a growth in spending on biscuits, cakes and other snack foods, linked to both the affordability and availability of such items alongside a general decline in home cooking [7]. Despite this, there has been an increase in television cooking programs globally, and programs focused specifically on baking, for example, The Great Australian Bake Off (Foxtel) and Zumbo′s Just Desserts (Seven network), which represents baking as fun rather than work, appealing to both men and women, where “ready-made” and pre-prepared products are shunned [8]. Currently, flour sales are several months ahead of projections, with many consumers locally and internationally baking bread and other items during isolation due to the COVID-19 pandemic [9]. In addition to this, new products have entered the flour segment of the supermarket, providing a diversified offering for consumers.

This study aimed to provide a cross-sectional analysis of flour for home baking, highlighting the nutrition composition, benefits of whole grain (wholemeal) flour and the variety of novel products within this category.

## 2. Materials and Methods

An audit of flour products was conducted in February 2020, in four major supermarkets in metropolitan Sydney (Aldi, Coles, IGA and Woolworths). Collectively, these supermarket chains make up more than 80% of total Australian market share, and were chosen in preference to smaller, independent grocery stores in an attempt to reflect food choices that the majority of Australians are faced with during food shopping [10]. This recognised process has been outlined in previously published research [4]. Smartphones were used to photograph the front, back and side labels of all available flour products present at each store both in the baking and health food aisle. Ethics approval was not required for this study. However, permission to take photos was obtained by supermarket store managers prior to data collection. The following information was collected for each product: ingredient lists, Nutrition Information Panel (NIP) per serve and per 100 g, percentage of whole grain, health and nutrition related claims, additional logos, endorsements and country of origin. Data from photographs were transcribed into a Microsoft^®^ Excel^®^ spreadsheet (Version 2013, Redmond, Washington, USA) for analysis. Products were classified according to the descriptions of the types of flour outlined in Table 1, including flour from grains like wheat, corn, rice, pseudograins including buckwheat and quinoa, and novel products, for example fruit and vegetable products. Flours with added canola or vegetable oil (e.g., pastry mix) and added sugar (e.g., scone/brioche bread mix) were excluded. A supplementary internet search was conducted through supermarket websites and identified manufacturer websites using key words to ensure that all products were captured. It is worth noting that data collection took place prior to COVID-19 panic purchasing in Australia, which resulted in increased sales of flour by 156% and 170% for bread-making flour mixes in the March–April 2020 period according to Nielsen data [9].

Eligibility for products to make nutrition content claims was assessed, in line with FSANZ and the Grains & Legume Nutrition Council’s (GLNC) Code of Practice for Whole Grain Ingredient Content Claims (The Code) [11]. Eligibility for registration with The Code was used to classify products as ‘whole grain’ or ‘refined grain’ to allow for nutrient comparison between whole grain and refined grain flours. Based on The Code eligibility criteria, products were classified as ‘whole grain’ if the flour contained more than or equal to 8 g of whole grain per manufacturer serve and ‘refined grain’ if the flour contained less than 8 g of whole grain per manufacturer serve. Non-grain flours derived from coconut, fruit/vegetables, legumes, hemp, tiger nut and tapioca starch were excluded from the whole grain and refined grain nutrient comparison. A second, independent reviewer checked data for any inconsistencies and errors.

### Statistics

All data were checked for normality using the Shapiro–Wilk test (IBM SPSS^®^, version 25.0, IBM Corp., Chicago, IL, USA) and the median and range were presented. A Kruskal–Walis one-way ANOVA with Bonferroni correction for multiple test (IBM SPSS^®^, version 25.0, IBM Corp., Chicago, IL, USA) was used to identify differences (1) in the five main flour categories (plain, self-raising, bread-making mixes, gluten-free and other refined flour products) and (2) the within-group differences across the nutrient criteria. A Mann–Whitney U test was used to examine the differences between nutrients in whole grain and refined grain flour, defined according to each product′s eligibility for registration with The Code (≥8 g whole grain per manufacturer serve) [12]. Missing values for dietary fibre were expected, as these are not included on packaging unless claimed, so this was analysed separately in both instances.

## 3. Results

Overall, 130 products were collected (26 plain wheat flour, 12 self-raising wheat flour, 17 plain wholemeal flour, 4 wholemeal self-raising flour, 20 bread-making mixes, 20 other refined grain (including corn and rice flour), 17 gluten-free, 3 legume, 4 fruit/vegetable, 4 coconut and 3 other non-grain (including hemp and cricket flour) products). As the number of whole grain products was small, the analysis of the main categories presented in Table 2 examines the differences between plain and self-raising flour with white and wholemeal flour varieties categorised together. 

Although flour appears a homogenous group, there were significant differences in the nutrient criteria across all five main flour categories, except for saturated fat (Table 2). In order to identify the specific differences, a between-category analysis of the five main flour categories provided further information differentiating products (Appendix A). In terms of energy, there was a significant difference between bread-making mixes and other refined grain flour (*p* 0.001), plain flour (*p* 0.003) and gluten-free flour (*p* 0.032) as this category was lower in energy. The gluten-free flour protein content was lower than most other flour types and was significantly different to the plain flour (*p* < 0.001), self-raising flour (*p* < 0.001), and the bread-making mixes (*p* 0.001). Protein also differed between the other refined grain flour and plain flour (*p* < 0.001) and self-raising (*p* 0.007) as the products in this group were lower in protein than wheat flour. Difference in fat reached significance only between gluten-free and plain flour (*p* 0.016) in the between-category analysis. Carbohydrate was lower in bread-making mixes compared with plain flour (*p* <0.001), gluten-free flour (*p* <0.001) and these differences also existed between other refined grain, which was higher in carbohydrate (~10 g/100 g higher) than plain flour (*p* 0.007). Sugars were lowest in other refined grain flour products and were significantly different compared to plain (*p* 0.005) and self-raising flour (*p* 0.015). Dietary fibre was lower in gluten-free flour, resulting in significant differences compared with plain flour (*p* 0.002), self-raising (*p* 0.027) and bread-making mixes (*p* 0.004). Whereas self-raising flour was the highest in sodium, plain flour and other refined grain flour types were the lowest in sodium, with significant differences between self-raising flour (*p* < 0.001; *p* < 0.001), bread-making mixes (*p* 0.003; *p* 0.035) and gluten-free varieties (*p* < 0.001; *p* 0.004) ranked third highest in sodium. 

Although ranked second highest in sodium, bread-making mixes contained less than half of the sodium found in self-raising (white and wholemeal) varieties (*p* 0.022). When the main flour categories were reclassified as either ‘whole grain’ or ‘refined grain,’ as previously outlined, wholemeal made up 19% of the category and included products based on wheat, buckwheat, millet, rye, khorasan, spelt and quinoa which met the definition of whole grain (including wholemeal bread-making mixes, *n* = 4). Table 3 compares the nutrient composition of whole grain and refined grain flours.

Overall, 25 grain-based flours were considered ‘whole grain’ including 17 plain wholemeal flours, 4 wholemeal self-raising flours and 4 wholemeal bread-making mixes. As shown in Table 3, whole grain products were significantly lower in energy, carbohydrate and higher in protein, fat, saturated fat, and sugars compared with refined products. Whole grain flours were 2.8 times higher in dietary fibre compared with refined grain flour products. There was no difference in sodium. However, a wide range in values for each category was apparent.

Fourteen individual novel flour products across nine categories were included in this audit (Table 4), making up 11% of total products—many of them new to the market. Some products were not specifically called flour, and instead used the word ***powder*** as a descriptor. Bolded figures in Table 4 represent particularly high values for the nutrient within this category of novel flour. Insect powder made from crickets, hemp seed and legume flour were the highest in protein. In comparison to plain flour, which contains ~10 g/100 g protein, products were two to six times higher. Broccoli, coconut, sweet potato and hemp flour were the highest in dietary fibre, with two to five times that of wholemeal flour. Tiger nut, cricket powder and coconut flour were far higher in both fat and saturated fat, both nutrients that are typically found at low levels in grain-based flour products.

Table 5 ranks the frequency of front-of-pack claims in the flour category with ‘No artificial colours/flavours/preservatives’ the most common claim made on packaging, featuring on more than 1/3 of the total category. Gluten free was also featured on more than 25% of products, important for those needing to comply with therapeutic diets. Dietary fibre claims were also prevalent in this category, with just over 20% of products making this claim. 

The majority of flours (76%, *n* = 99) were made, grown or produced in Australia including all bread-making mixes (*n* = 20), all wholemeal wheat flours (*n* = 8), 88% of gluten-free flours (*n* = 15) and 87% of the white wheat flours (*n* = 33). Other flours were sourced from a range of countries including Italy, China, India, Thailand, Canada, South Africa, Sri Lanka, Vietnam and Peru. Interestingly, the cricket powder specifically promoted the fact that the crickets were born and bred in Sydney, Australia, on their labelling, which added a provenance story to their sustainability platform.

## 4. Discussion

Although baked goods like bread, cakes and biscuits may often be purchased outside of the home, the supermarket flour category for home baking has increased in number and range of products in a relatively short period of time. According to the Mintel product database, there has been a 77% increase in Australia in product numbers (from 22 to 39 products) captured between September 2018 and January 2020. Global launches increased in the same period by 8% from 1453 to 1568 products (a difference of 115 products) [13]. In this analysis, there were significant differences in all nutrients (except saturated fat) across the five main flour categories. Products marketed as gluten free were lower in both dietary fibre and protein, which makes naturally gluten-free products made of legumes (see Table 4), buckwheat (with 10 g dietary fibre and 12.4 g protein) and quinoa (with 6.8 g dietary fibre and 10.9 g protein), a potential better choice. 

‘Plant protein’ and ‘sustainability’ are major themes in food marketing in this new decade and this may also be driving continued innovation within flour and baked products. Novel options in flour perhaps linked with ‘reconnecting [consumers] with the sources of our food’ and sustainable practices within the food supply system [14], for example vegetable products made from potential waste product.

Less than 20% of the flour products were considered whole grain. In diets, bread and breakfast cereal are known to be the main two food categories contributing whole grain [15]. However, swapping to wholemeal flour in baking would more than double dietary fibre in baked goods. In Australia, as is the case elsewhere in the world, whole grain intake is low, with a secondary data analysis from the Australian Health Survey recording a median intake for children at 16.5 g per day, and adults at 21.2 g/day—both less than half of the established Daily Target Intake (DTI) of 48 g per day for adults, and between 32 and 40 g per day for children [11,16,17]. This is also true elsewhere, as many countries fall short, such as UK [18] and USA [19], but also in Europe (France [20]; Italy [21]), and in Asia (Malaysia [22]; Singapore [23]). Equally, a large body of evidence points to the benefits of increasing dietary fibre and its role in reducing chronic disease risk, yet most Australians fall short, with more than half of children, and more than 70% of adults not meeting their respective targets [24]. Habits formed at home are known to be powerful in influencing habitual intake, and this is also true for whole grain [25]. According to the FSANZ Food Composition Database, wholemeal wheat flour is also naturally more nutrient dense than white flour, with more calcium, chloride, fluoride, phosphorus, potassium, selenium, and zinc, twice as much magnesium and manganese, three times as much niacin, four times the folate, and eight times the amount of vitamin E [26]. 

Unlike other nutrients, whole grain claims are not regulated by FSANZ in Australia and New Zealand, rather, they fall under a voluntary Code of Practice for Whole Grain Ingredient Content Claims (The Code), introduced in 2013 by GLNC to encourage evidence-based promotion of whole grain foods. GLNC utilises audits of grain-based foods to monitor the operation of The Code and provide feedback to industry [15]. While 27% of eligible flour products from this audit were registered with The Code, there remains opportunities to explore alternative whole grain products for example, buckwheat and millet. Unlike other grain food segments, declaring the percentage whole grain is not an issue for the flour category as most products are either 100% whole grain or they are refined. There were instances where whole grain percentage was not reported, and these were classified as refined products, a potential limitation of the voluntary system. Bread-making mixes may be the only subcategory where smaller percentages of whole grain are declared, as these products tended to be refined with added seeds and grain to make a multi-grain product, which does not fit the definition of whole grain. Recent research regarding whole grain in food has been giving attention to milling methods, with micronized flour milling to assist with texture and sensory profiles to encourage consumption [27], while other research has focused more on glycaemic control and outcomes relevant in Type 2 Diabetes Mellitus [28,29], with concerns about the degree of milling. These issues may need to be resolved to help progress discussions regarding the definition of whole grain foods [30].

With the sodium content of diets as the leading issue in relation to the Global Burden of Disease [31], the significant difference in the level of sodium between self-raising flour and plain flour categories is a reminder for home bakers and health care professionals that the addition of sodium bicarbonate (or sodium hydrogen carbonate) and cream of tartar (potassium hydrogen tartrate) for chemical leavening is a contribution to the intake of this public health-sensitive nutrient to the overall diet. In bread-making, yeast is primarily used for leavening, and salt plays a different role in controlling the yeast, and adding flavour, although this category contained less than half of the sodium found in self-raising (white and wholemeal) varieties. Although bread contributes sodium in the Australian diet, this food is also a key vehicle for iodine fortification of the food supply. The Food and Health Dialogue target for bread recommended that sodium be less than 400 mg/100 g and from a study of loaf breads published in 2018, over half (54%) of all loaves complied with this target, having reduced by 11% since 2014. It is worth noting that the Healthy Food Partnership, an initiative of the current Australian Government, may suggest an even lower target of 380 mg/100 g for sodium [4]. 

There are a range of alternative flours launched locally and globally, made from legumes, ancient grains, indigenous wheat and insects featuring extra protein and dietary fibre, appealing to the ‘natural’ consumer [32]. Many of these have moved from specialty areas of the supermarket focused on special diets (known as the health food aisle) to the general baking aisle. In this audit, we identified fourteen novel flour products, with seven relatively new to the market. Interestingly, several products in this novel category are referred to as ‘flour’ on pack, despite falling outside of the specific definition of a flour according to FSANZ. For example, cauliflower, coconut, green banana and tiger nut are referred to as ‘flour’, but are not derived from cereals, legumes or other seeds [33]. To encourage clear and consistent product labelling, it would be more appropriate to classify these products as powders, in line with similar fruit and vegetable products identified in this audit. Many products in the novel category would behave very differently to wheat-based flour in baking, absorbing far more liquid, providing protein perhaps, yet no gluten for the structure of the product or other attributes, making them unsuitable for totally replacing grain-based flour in traditional baking. In this respect, these products may be used to augment wheat flour in baking, providing some very positive nutritional attributes, but may not fully replace wheat-based flour. 

Coconut flour, derived from the dried, ground flesh, absorbs a great deal of liquid in baking, and recipes would need to be adjusted. While the product is very high in dietary fibre, this may be the only nutritional attribute worth promoting due to the higher fat, saturated fat and sugars. Blended flour products previously on the market containing a combination of wheat, millet, oat and coconut flours were no longer available on shelf during this audit. This perhaps points to problems in exchanging wheat flour for the blended products containing coconut, as they suggested, ‘cup-for-cup’ exchange on the packaging, and this may not have been precise enough for home bakers. Coconut flour currently on the market suggests on the packaging that 1C of wheat flour can be replaced by 1/3C of coconut flour and extra liquid or egg may be needed to compensate, and in addition to this, the overall volume of the mix would be affected.

Among the novel flour products, broccoli, sweet potato and hemp flour were also very high in dietary fibre, even higher than legume flour. Legume flour has been investigated for suitability in baking [34,35], and when mixed with other grains to make bread, biscuits and other baked products. The amino acid profile of legumes is known to be complementary to cereal grains, improving the nutritional properties. However, legume varieties with lower levels of antinutrients would be preferred [36]. Lentils, chickpeas and peas have been trialed, with acceptable results where chemical leavening is also used [34]. High-quality bread is more difficult to produce with legume flour, as the protein in legumes do not form a gluten network and reduce the ability of the wheat proteins in forming visco-elastic properties, limiting the incorporation of air and therefore gas retention, impacting on the structure of the crumb and overall texture. An 80–20% wheat–lentil flour bread has been tested with success, with researchers commenting on the favourable carbohydrate profile and prebiotic functionality, which possibly assists with overall bowel health [35]. The competitive nature of legume flour in a bread would be more detrimental in a wholemeal bread product, where the bran also has an impact, slicing through gluten strands and affecting the overall structure of the gluten matrix. So where protein and dietary fibre content would be improved through the addition of legume flour, the wheat flour would almost certainly need to be refined, although Brescianai et al. comments that studies often do not specify any details on the type of base flour, making comparisons between studies difficult [34] and conclusions regarding the optimal formulation an opportunity for future research.

The products using ‘powder’ as a descriptor included broccoli, sweet potato, cricket and hemp products. Broccoli powders have recognised potential as a natural food supplement. Belonging to the family Cruciferae and the genus *Brassica*, it is rich in vitamins (C, A and folic acid) and minerals (potassium, phosphorus, calcium and sodium), and a range of other protective compounds. A disadvantage of this crop is that as much as 70% is commonly discarded in the field [37], and as a result, recovery and bioconversion of vegetable residue has encouraged potential uses for broccoli waste. Interestingly, the dietary fibre content in studies comparing florets, leaves and stalks ranged from 11.65 to 15.75 g/100 g dry weight, highest in the stalk, but these figures are far lower than the 44 g/100 g reported on pack via this audit. The resulting broccoli powder was also reportedly high in fatty acids and amino acids, with only methionine in low concentration [37]. These qualities make the product suitable to add to soups or bakery products, although very few published papers are available supporting these alternate uses. Similarly, cauliflower flour, where 45–60% of the total weight of the raw vegetable is considered waste, has been used to produce a flour product that is high in dietary fibre and antioxidants [38]. In the case of cauliflower, the waste produces a foul odour on decomposition, so management of vegetable waste streams are important. The potential uses for cauliflower flour have been explored in biscuit making [38], and on-pack messages promote cauliflower flour as a direct replacement for grain-based flour, cup-for-cup, although this would need to be specifically tested in recipes. Other vegetable-based powder products made from broccoli and sweet potato are suggested as additions to meals and drinks in a more general way, without supplying any instructions on how to make these additions, further encouraging a trial-and-error approach which may deter some consumers.

Bananas are the second most popular global crop, after citrus, although large portions of a crop may be rejected if they are immature, cracked or irregular [39,40]. Green banana flour was developed in Queensland, Australia, by farmers wanting to avoid waste of unripe green bananas [41]. They found that the banana naturally formed a flour-like product when dried and as the starch had not yet had time to convert to sugars, the flour product tastes like bran rather than banana, and is high in resistant starch [40]. The packaging of this product provided specific instructions on how to use in place of wheat flour in baking and also how to add chemical leavening agents. As green banana flour absorbs more water than wheat flour, the exchange is ¾ C banana flour for 1 C of wheat flour. As with legume flour, in a product like bread, green banana flour could only be used in replacement of ~30% of wheat flour, increasing resistant starch and minerals such as phosphorus, potassium, magnesium and calcium [40,42]. Many gluten-free breads are of lower quality in terms of texture, crumb and mouthfeel [42], in addition to being lower in protein and dietary fibre [4]. As green banana flour is gluten free, it has the potential to improve the dietary fibre content of gluten-free bread [42]. Green banana flour has also be used in creation of pasta and noodle products, but they need to withstand the high cooking temperatures when boiled in water [39].

Despite its name, tiger nut flour is derived from a starchy root rather than a nut, although it was not as high in carbohydrate as the green banana flour. Tiger nut flour was high in fat and saturated fat, and this gluten-free flour alternative was promoted as great for baking sweet or savoury treats, with twice as much dietary fibre as wholemeal flour. In the scientific literature, it has been tested in biscuits [43], noodles [44] and pasta with ~40% substitution of tiger nut flour [45]. Tests in bread were combined with legumes, making it more difficult to assess the value or physico-chemical effects of the tiger nut flour in this food [46].

New Nutrition Business has identified plant protein as a key driver of food choice in recent years and this trend is apparent in this food category [47]. Hemp seed with low delta 9-tetrahydrocannabinol varieties of *Cannabis sativa* were approved as a food by FSANZ in April 2017 and both the seeds and the oil are used in Europe, Canada and the USA [48]. Containing high levels of protein, polyunsaturated fatty acids, particularly omega-3 fatty acids and dietary fibre, the seed is well suited as a flour. However, it should be noted that it was the only novel product not specifically suggested as an addition to baking or baked goods. The suggested use, in smoothies, shakes or nut butters, all of which are uncooked products, may be important in preserving the fragile polyunsaturated fats in the hemp seed, as these would be altered through a baking process. Despite this, a number of studies support the addition of hemp in bread products with enhanced nutrition, acceptable colour and sensory qualities [49,50]. In alignment with the sustainability theme, insect flour made from crickets was among the highest in protein, fat and saturated fat in this audit. While undoubtedly sustainable, this product is very dependent on consumer views [51,52]. Like the vegetable flour products, cricket flour, could only be added to baked goods in small quantities and the product packaging suggests pancakes, cakes, biscuits, homemade bars and bread. A range of food products have been evaluated in the literature including crackers [53], buns [54], muffins [51] and bread [55], with generally positive acceptance at low levels of substitution.

Strengths of this study include its comprehensive nature and, to our knowledge, it is the first study that has compared the range of flour products and novel entrants to the market. While all efforts were made to capture the category in its entirety, differences may exist between geographic areas. In addition to supermarkets, there are a range of bulk food stores selling alternative grain flours including teff, sorghum and millet. However, these are not often contained within packaging and were excluded from this analysis (*n* = 31). As previously stated, reporting of dietary fibre and whole grain within the ingredients and in the Nutrition Information Panel is not mandatory in the absence of an on-pack claim, so this information was not always declared, and thus there was some missing data. Finally, we did not conduct an independent nutrition analysis of products and were reliant on manufacturer information to draw comparisons between product categories. 

## 5. Conclusions

Flour for home baking has typically been derived from wheat, rye, corn and rice but in recent years, new products have emerged within the category. Alternative and ancient grains, pseudograins and novel products from insects, fruit, vegetables and coconut can now be found in either the home-baking or health food aisle of supermarkets. Although products are often referred to as ‘flour,’ they may have vastly different nutritionals and baking properties, and some fall outside of the FSANZ definition for flour, as they are not derived from cereals, legumes or seeds. As a result, the key issue for this segment may eventually be of a regulatory nature. In any case, the mainstay of plain refined wheat flour remains, with a clear opportunity for further promotion of whole grain flour from a variety of grains or pseudograins (wheat, buckwheat, millet, spelt, quinoa and others). Although less than 20% of products in this analysis were whole grain, a simple swap to wholemeal flour in suitable recipes would more than double dietary fibre in home-baked products. The packaging claims on novel products are compelling, and for mainstream grain products, improved on-pack information, clearly articulating the benefits of dietary fibre, including descriptions of how to exchange white for whole grain, could attract more consumers. The added directions for consumers may in turn help flour manufacturers and health professionals alike encourage the use of whole grain and higher-fibre grain-based flour products in baking.

## Figures and Tables

**Table 1:** Classification of flour categories.

Category	Description of Types
White, plain	Derived from ground wheat grain (endosperm) with bran and germ removed. No added leavening agents.
White, self-raising	Derived from wheat grain (endosperm) with bran and germ removed with added leavening agents.
Wholemeal, plain	Derived using the whole wheat (whole grain) or other grain; contains endosperm, bran and germ. No added leavening agents. Includes pseudograins and ancient grains (e.g., quinoa, buckwheat, spelt, teff and khorasan).
Wholemeal, self-raising	Derived using the whole wheat (whole grain) or other grain; contains endosperm, bran and germ with added leavening agents.
Bread-making mixes	Combination of high-protein flour types, may contain added gluten; may contain added grains and seeds; may contain added vitamins or minerals.
Gluten free	Includes flours marketed as gluten free; contains maize and/or tapioca starch as the primary ingredient.
Other refined grain	Includes corn and/or rice flour.
Coconut	Derived from the flesh of a mature coconut, dried, and ground to a fine powder.
Legume	Derived from dried, ground (uncooked) legumes, e.g., chickpeas, red lentils, and soybean.
Other non-grain	Includes novel flour derived from dried, ground insects; from roots, e.g., tiger nut flour; seeds, e.g., hemp.
Fruit/vegetable	Novel flour or powder products from potential fruit and vegetable waste, malformed product, skins or other offcuts.

**Table 2:** Nutrient composition of the main flour categories per 100 g (median and range).

Nutrient Criteria	Plain Flour (White and Wholemeal) (*n* = 43)	Self-Raising Flour (White and Wholemeal) (*n* = 16)	Bread-Making Mixes (*n* = 20)	Gluten Free (*n* = 17)	Other Refined Grain (*n* = 20)	*p*-Value *
Energy (kJ)	1470 (1390–1606)	1440 (1390–1579)	1058 (921–1530)	1476 (644–1554)	1482 (1390–1615)	0.001
Protein (g)	10.9 (0–15.9)	10.0 (9.2–14.2)	9.0 (5.1–12.7)	2.3 (1.0–5.0)	6.6 (0.4–10.9)	<0.001
Total Fat (g)	1.7 (0.8–5.9)	1.5 (1.1–4.4)	1.7 (0.8–4.3)	1.0 (0–4.7)	1.2 (0–8.2)	0.016
Saturated Fat (g)	0.5 (0–1.0)	0.4 (0.2–1.0)	0.5 (0.1–1.0)	0.4 (0–2.0)	0.6 (0–1.0)	0.839
Carbohydrate (g)	70.6 (58.5–89.3)	70.0 (62.7–76.2)	47.9 (39.8–74.6)	84.3 (36.5–90.0)	76.9 (57.7–89.2)	<0.001
Sugars (g)	1.1 (0.1–7.7)	1.5 (0.3–3.3)	0.5 (0–5.1)	1.0 (0.1–12.0)	0.8 (0–2.0)	0.001
Dietary Fibre (g)	3.6 (0.5–10.7)	3.3 (2.9–8.8)	4.1 (2.1–10.7)	1.0 (0.7–7.1)	3.0 (0–10.0)	0.001
Sodium (mg)	5.0 (0–228.0)	690.0 (320.0–890.0)	325.0 (1.1–1000.0)	283.0 (5.0–775.0)	5.0 (0–45.0)	<0.001

* Kruskal–Walis one-way ANOVA with Bonferroni correction 95% CI.

**Table 3:** Comparison of nutrients in whole grain and refined grain flours (per 100 g) (median and range).

Nutrient Criteria	Whole Grain * (*n* = 25)	Refined Grain ** (*n* = 91)	*p*-Value
Energy (kJ)	1420 (921–1606)	1470 (644–1615)	0.012
Protein (g)	11.3 (7.6–14.6)	9.0 (0–15.9)	<0.001
Total Fat (g)	2.4 (1.6–5.9)	1.4 (0–8.2)	<0.001
Saturated Fat (g)	0.7 (0.3–1)	0.4 (0–2.0)	0.05
Carbohydrate (g)	63.3 (39.8–71.0)	72.7 (36.5–90)	<0.001
Sugars (g)	1.6 (0.4–7.0)	1.0 (0–12.0)	0.004
Dietary Fibre (g)	8.8 (5.7–10.7)	3.1 (0–10)	<0.001
Sodium (mg)	7.0 (1–685)	8.0 (0–1000)	0.743

Mann–Whitney U test 95% CI. * Based on eligibility for registration with GLNC’s Code of Practice for Whole Grain Ingredient Content Claims (≥8 g per manufacturer serve). ** Includes two flours that did not report percentage of whole grain ingredients.

**Table 4:** Nutrient composition of novel flours per 100 g reported on Nutrient Information Panel (mean).

Nutrient Criteria	Coconut	Legum	Cauliflower	Green Banana	Broccoli	Sweet Potato	Cricket	Tiger Nut	Hemp
	(*n* = 4)	(*n* = 3)	(*n* = 1)	(*n* = 1)	(*n* = 1)	(*n* = 1)	(*n* = 1)	(*n* = 1)	(*n* = 1)
Energy (kJ)	1809	1598	1480	1470	1030	1200	1760	1890	1700
Protein (g)	16.7	28	7.2	4	17.8	3.7	65.6	4	41.6
Total Fat (g)	14.6	11.4	1.9	1	3.3	2.1	16	26	20
Saturated Fat (g)	13.5	2	1	1	1	1.3	5	6	1
Carbohydrate (g)	40.2	34.3	72.2	77.1	14.4	51.7	1	43.5	5.5
Sugars (g)	14.2	3.9	15.8	2.5	13.6	19	1	15	3.5
Dietary Fibre (g)	29.9	13.4	7	7.8	44.8	22.5	6	15.5	20.1
Sodium (mg)	29	4	310	5	364	20	435	40	5

**Table 5:** Rank order of on-pack claims.

Nutrition/Health Claim	Total Products Making Claim (%)
No artificial colours/flavours/preservatives	37.9% (*n* = 49)
Gluten free	26.9% (*n* = 35)
Dietary fibre	22.3% (*n* = 29)
Non-genetically modified	18.5% (*n* = 24)
Vegetarian/vegan	16.9% (*n* = 22)
Dairy free	15.4% (*n* = 20)
Protein	14.6% (*n* = 19)

## Appendix A

**Table A1:** Grain-based flours post hoc comparison *p* values (CI 95%).

Nutrient Criteria	Product Category		Product Category				
		Direction	Plain Flour (White and Wholemeal)	Self-Raising Flour (White and Wholemeal)	Bread-Making Mixes	Gluten Free	Other Refined Grain
Energy (kJ)	Bread-making mixes	*Lower*	*p* 0.003			*p* 0.032	*p* 0.001
Protein (g)	Gluten free	*Lower*	*p* < 0.001	*p* < 0.001	*p* 0.001		
	Other refined grain	*Lower*	*p* < 0.001	*p* 0.007			
Total Fat (g)	Gluten free	*Lower*	*p* 0.016				
Carbohydrate (g)	Bread-making mixes	*Lower*	*p* < 0.001			*p* < 0.001	
	Other refined grain	*Higher*	*p* 0.007				
Sugars (g)	Other refined grain	*Lower*	*p* 0.005	*p* 0.015			
Dietary Fibre (g)	Gluten free	*Lower*	*p* 0.002	*p* 0.027	*p* 0.004		
Sodium (mg)	Plain flour	*Lower*		*p* < 0.001	*p* 0.003	*p* < 0.001	
	Other refined grain	*Lower*		*p* < 0.001	*p* 0.035	*p* 0.004	
	Bread-making mixes	*Lower*		*p* 0.022			
